# Toward SDG 3 in a developing nation: the role of health expenditure, financial development, and population growth in shaping health outcomes

**DOI:** 10.3389/fpubh.2025.1657624

**Published:** 2025-09-03

**Authors:** Xiaohan Xiang, Shengwei He, Aysha Abid

**Affiliations:** ^1^Guangzhou College of Technology and Business, Guangzhou, China; ^2^Department of Political Science, Minhaj University, Lahore, Pakistan

**Keywords:** life expectancy, infant mortality, under-five mortality, economic indicators, error correction model (ECM), health policy in Bangladesh

## Abstract

This study examines how macroeconomic and demographic factors influence public health outcomes in Bangladesh from 2000 to 2022, using the Autoregressive Distributed Lag (ARDL) approach and the associated Error Correction Mechanism (ECM). The estimated coefficients represent marginal effects, which indicate the absolute change in each health indicator for a one-unit change in the explanatory variables, holding other factors constant. The analysis focuses on three health indicators: life expectancy at birth, infant mortality rate, and under-five mortality rate, and four explanatory variables: public health expenditure, GDP per capita, domestic credit to the private sector, and population growth rate. Results show a stable long-run relationship among the variables. Higher public health expenditure, GDP per capita, and financial development are generally associated with improved health outcomes, while rapid population growth is linked to poorer child health. The ECM results indicate that when short-term fluctuations occur, health outcomes gradually return to their long-term path. The Error Correction Term (ECT) measures the speed of this adjustment and shows how quickly equilibrium is restored after a disruption. These findings suggest that while economic growth and financial development can support better health outcomes, their impact depends on managing demographic pressures and improving the efficiency of health spending. Coordinated fiscal, financial, and population policies are essential to sustain health gains and advance Bangladesh’s progress toward Sustainable Development Goal 3 (SDG 3).

## 1 Introduction

Health is a fundamental pillar of human development and a vital indicator of a nation’s progress. In low- and middle-income countries (LMICs) like Bangladesh, improving health outcomes remains a central policy goal, especially under Sustainable Development Goal 3 (SDG 3): ensuring healthy lives and promoting well-being for all ages (1).

Bangladesh has made significant strides in health over the past two decades. Life expectancy increased from 65.2 years in 2000 to 72.4 years in 2022, while infant and under-five mortality rates dropped from 64 and 84 to 24 and 29 per 1,000 live births, respectively (2). These gains reflect expanded public health efforts, donor support, and private sector involvement. However, disparities in access, quality, and outcomes persist, particularly between urban and rural populations and across socioeconomic groups (3). Understanding the macroeconomic and demographic drivers of health is critical for targeted policy. Public health expenditure (PHE), when effectively allocated, enhances service delivery and reduces mortality (4). Yet, Bangladesh’s health spending remains relatively low at around 2.4% of GDP (5).

GDP per capita, a core economic indicator, reflects national income levels and household capacity to invest in health and nutrition. The Preston Curve illustrates that rising income is generally associated with better health outcomes (6), a trend observed in Bangladesh as well. However, income distribution and service equity are also crucial for translating growth into health improvements.

Financial development, proxied by domestic credit to the private sector, enables investment in health-related infrastructure and helps households manage health shocks (7). While financial access in Bangladesh has improved, credit gaps remain, especially in underserved areas. Population growth rate (POPGR), meanwhile, presents dual implications. A young population can fuel economic growth, but high growth also strains health systems and reduces per capita service access (8).

**Figure 1:**
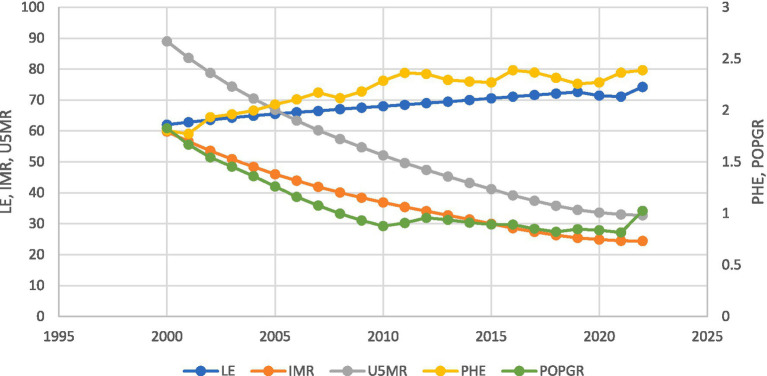
Trends in key macroeconomic and health indicators in Bangladesh (2000–2023), including Public Health Expenditure (PHE), Population Growth Rate (POPGR), Life Expectancy at Birth (LE), Infant Mortality Rate (IMR), and Under-5 Mortality Rate (U5MR).

Figure 1 demonstrate significant improvements across key health and economic indicators. Life Expectancy at Birth (LE) rose steadily from 62.0 years in 2000 to 74.3 years in 2023, signaling advancements in healthcare infrastructure, nutrition, and living standards. In parallel, Infant Mortality Rate (IMR) fell sharply from 59.8 to 24.4 deaths per 1,000 live births, and Under-5 Mortality Rate (U5MR) for males dropped from 89 to 32.7, reflecting enhanced maternal and child health services and expanded immunization coverage. On the fiscal side, Public Health Expenditure (PHE) as a share of GDP increased from 1.80% in 2000 to 2.39% in 2022, indicating a strengthened public commitment to health sector funding (9, 10). Meanwhile, the Population Growth Rate (POPGR) declined from 1.83% to just over 1.02%, suggesting effective demographic management and progress toward a stable population structure.

Despite the relevance of these variables, existing empirical studies on Bangladesh often examine them in isolation or use basic models that do not distinguish long-run from short-run effects (11). To address this gap, this study applies the Autoregressive Distributed Lag (ARDL) bounds testing approach and the associated Error Correction Mechanism (ECM) to analyze both long-run equilibrium and short-run dynamics among public health expenditure, GDP per capita (US$), financial development, and population growth, in relation to three core health outcomes: life expectancy at birth, infant mortality rate, and under-five mortality rate.

Focusing on Bangladesh provides context-specific insights for a country that has made notable health progress but continues to face systemic challenges. These findings can inform policymakers in similar LMIC contexts seeking to design integrated strategies for sustained health improvement, especially in a post-COVID-19 recovery phase.

Although prior studies have examined the influence of economic and demographic factors on health outcomes in Bangladesh and other low- and middle-income countries, most have done so with important limitations. First, many focus on a single health indicator, such as life expectancy or infant mortality, rather than considering multiple complementary health outcomes together (12). Second, existing works often apply static models or simple correlations, which fail to distinguish between short-run fluctuations and long-run equilibrium relationships (13). Third, the role of financial development, particularly domestic credit to the private sector, has been largely overlooked in the Bangladesh context, despite its potential to shape healthcare access and infrastructure investment (14). Finally, few studies explicitly account for structural breaks arising from major events such as the COVID-19 pandemic, which may significantly alter health–economy linkages (15).

This study addresses these gaps by integrating multiple health indicators into a unified framework and applying the Autoregressive Distributed Lag (ARDL) bounds testing approach combined with an Error Correction Mechanism (ECM) to distinguish short-run from long-run effects (16). The inclusion of a structural break dummy allows the analysis to capture potential disruptions caused by the COVID-19 pandemic. This methodological combination provides a more comprehensive and dynamic assessment of the interactions between public health expenditure, economic performance, financial development, population growth, and key health outcomes in Bangladesh, generating insights that are directly relevant for both national policy and the wider literature on health economics in developing countries.

## 2 Literature review

Understanding the macroeconomic and demographic determinants of health outcomes has been central to research in development economics and public health. Existing literature generally agrees that economic growth, public health spending, financial sector development, and demographic trends influence health indicators such as life expectancy, infant mortality, and under-five mortality. However, comprehensive dynamic analyses of these relationships in the context of Bangladesh remain limited (17).

Economic development, typically measured by GDP per capita, is widely recognized as a major determinant of health. The seminal Preston Curve demonstrates a positive relationship between income and life expectancy, particularly in low-income countries (6). In Bangladesh, Acheampong and Opoku (18) found that rising per capita income improved nutrition, healthcare access, and sanitation, contributing to lower mortality and longer life expectancy. However, Mdingi and Ho (19) note that these gains are not automatic; without equitable income distribution, economic growth can fail to produce proportional improvements in health outcomes. This suggests that the growth–health relationship may be conditional on broader social and policy factors.

Public health expenditure (PHE) has consistently been associated with better health outcomes. Globally, higher government spending on health correlates with lower mortality and longer life spans (20). In South Asia, Owusu et al. (21) show that sustained investment in health infrastructure reduces infant and maternal mortality, while Rajia et al. (22) link increased PHE in Bangladesh to improved child survival rates. Yet, other studies caution that inefficient allocation, weak governance, and delays in implementation can reduce the impact of health spending (23). Moreover, Bangladesh’s health expenditure has remained below 3% of GDP, raising concerns about both its sufficiency and its capacity to meet population needs (24).

Financial development, often measured by domestic credit to the private sector, is another factor influencing health outcomes. Greater access to credit can encourage investment in healthcare facilities, pharmaceuticals, and insurance schemes (25). In Bangladesh, the expansion of microfinance and banking services has lowered some financial barriers to care (26). However, Prodhan et al. (27) point out that these benefits remain unevenly distributed, especially in rural and underserved areas, limiting the overall health impact of financial sector growth.

Population growth presents a complex dynamic. While a younger population can increase economic productivity (28), rapid population growth can overburden healthcare infrastructure and reduce per capita access to services. In Bangladesh, Hossain et al. (29) report that rising population pressures have strained maternal and child health services, in some cases reversing progress in mortality reduction.

Methodologically, recent studies have increasingly applied dynamic models to health determinants. The Autoregressive Distributed Lag (ARDL) approach is particularly suitable for small samples and mixed integration orders, making it well-matched to Bangladesh’s data profile. Hidthiir et al. (30) applied ARDL in ASEAN to identify stable long-run relationships between macroeconomic factors and mortality, while Owusu et al. (21) found that health spending significantly influences infant mortality in the long term. Despite these advances, most studies still focus on a single health outcome, overlook the combined role of macroeconomic, financial, and demographic variables, and rarely account for structural breaks such as the COVID-19 pandemic.

This study addresses these gaps by examining three complementary health indicators within a unified ARDL–ECM framework that captures both short-run adjustments and long-run equilibrium relationships. It incorporates financial development alongside economic and demographic factors, while explicitly modeling the impact of structural breaks through a COVID-19 dummy. This approach provides a more comprehensive and context-specific assessment of health determinants in Bangladesh, contributing both to national policy relevance and to the broader literature on health economics in developing countries.

This study incorporates seven key variables to analyze the relationship between economic and health indicators in Bangladesh. PHE is defined as the percentage of GDP allocated to government spending on healthcare services and goods within a year. Domestic Credit to the Private Sector (DC) represents the allocated financial resources to private entities by financial institutions, also expressed as a percentage of GDP. Reflects the country’s economic output per person in current US dollars, serving as a measure of average income. POPGR indicates the annual percentage increase in the total population, capturing demographic dynamics. The health results variables include LE, which estimates the average life expectancy of a newborn under current mortality conditions; IMR, which denotes the number of infant deaths (under 1 year) per 1,000 live births; U5MR which measures the likelihood of a child dying before the age of five per 1,000 live births. These variables collectively provide a comprehensive view of the economic and demographic factors influencing health outcomes.

This study uses annual time-series data for Bangladesh from 2000 to 2022. The dependent variables include three key health indicators: life expectancy at birth (LE), infant mortality rate (IMR), and under-five mortality rate (U5MR). The core explanatory variables are public health expenditure as a percentage of GDP (PHE), GDP per capita in current US dollars (GDPPC), domestic credit to the private sector as a percentage of GDP (DC), and population growth rate (POPGR).

## 3 Data and estimation technique

The selection of variables is grounded in established empirical findings on the determinants of health outcomes in developing countries. Public health expenditure (PHE) is included because prior research demonstrates its significant role in improving health service delivery and reducing mortality, although its effectiveness depends on allocation efficiency and governance quality (20). GDP per capita (GDPPC) serves as a measure of national income and overall economic development, which the Preston Curve and subsequent studies link to improved nutrition, healthcare access, and life expectancy.

Financial development, proxied by domestic credit to the private sector (DC), is incorporated because access to credit can facilitate investments in healthcare infrastructure and services, thereby influencing health outcomes. Population growth rate (POPGR) is included due to its dual impact: while a growing young population can boost productivity, rapid population growth can strain health systems and reduce per capita service availability.

The dependent variables—life expectancy at birth (LE), infant mortality rate (IMR), and under-five mortality rate (U5MR)—are widely used summary measures of public health that capture both longevity and child survival. Using these three indicators together allows the analysis to reflect both general population health and vulnerable child health outcomes, an approach recommended in prior multi-indicator health studies.

Table 1 presents the definitions and sources of all variables used in the empirical analysis. For consistency, GDP per capita is uniformly labeled as GDPPC throughout the text and table.

**Table 1:** Variable descriptions.

Variable name	Symbol	Source	Definition
Public Health Expenditure (% of GDP)	PHE	World Bank	Share of GDP allocated to public health expenditures, including government spending on healthcare goods and services.
GDP per Capita (current US$)	GDPPC	World Bank	Gross Domestic Product divided by midyear population, expressed in current US dollars.
Domestic Credit to Private Sector (% GDP)	DC	World Bank	Financial resources provided to the private sector by financial institutions, as a share of GDP.
Population Growth Rate (%)	POPGR	World Bank	Annual percentage growth in the total population.
Life Expectancy at Birth (years)	LE	World Bank	Average number of years a newborn is expected to live under current mortality conditions.
Infant Mortality Rate (per 1,000 births)	IMR	World Bank	Number of deaths of infants under 1year per 1,000 live births in a given year.
Under-5 Mortality Rate (per 1,000 births)	U5MR	World Bank	Probability per 1,000 that a newborn will die before reaching age five, based on current age-specific mortality rates.

This study employs the Autoregressive Distributed Lag (ARDL) bounds testing methodology to examine the dynamic relationships between macroeconomic, financial, demographic variables, and health outcomes in Bangladesh from 2000 to 2022. The ARDL approach, developed by Pesaran, Shin, and Smith (16), is particularly well-suited to small sample sizes and can handle regressors that are stationary at level I(0) or first difference I(1), provided none is integrated of order two (I(2)).

The selection of the ARDL model over other dynamic models, such as the Vector Error Correction Model (VECM) or Generalized Method of Moments (GMM), is based on several methodological and data-related advantages. Unlike VECM, which requires all variables to be integrated at the same order (typically I(1)), ARDL accommodates a mix of I(0) and I(1) series. GMM, while powerful in dealing with endogeneity, is more appropriate for large panel datasets with multiple cross-sectional units and may not perform reliably with small time series data, such as the 23-year annual dataset used in this study. ARDL also allows for the inclusion of lags tailored to each variable, making it more flexible in capturing the short- and long-run dynamics characteristic of macroeconomic influences on health.

The Autoregressive Distributed Lag (ARDL) bounds testing approach, developed by, was selected for this study due to its several advantages. Unlike VAR or VECM models, ARDL can be applied when regressors are a mix of I(0) and I(1), which aligns with the integration order of our variables as confirmed by unit root tests. Furthermore, ARDL is well-suited for small sample sizes, a practical advantage given our 23-year dataset. The model allows simultaneous estimation of both short-run and long-run dynamics, offering more flexible lag structure selection. Diagnostic tests (serial correlation, heteroskedasticity, stability via CUSUM) confirmed that ARDL is appropriate and robust for the data under investigation.

Despite its strengths, the ARDL technique has limitations. It is sensitive to small sample bias, particularly if too many lags are included relative to the sample size. Careful model selection using criteria such as the Akaike Information Criterion (AIC) is therefore necessary. Additionally, while ARDL can tolerate some degree of endogeneity, it does not fully address multicollinearity among regressors, which can bias coefficient estimates. These considerations were addressed by pre-testing for unit roots and correlation diagnostics before model specification.

Initially, the ARDL cointegration method was employed, commencing with a VAR analysis to determine the appropriate lag length of the chosen variables. The appropriate lag length was determined using Akaike’s information criterion (AIC), subsequently applying the ARDL cointegration technique. The general form of model of study is study is shown in Equation 1.


(1) H0=f(PHE,GDPPC,POPGR,U5MR,IMR,LE,DC)

The ARDL cointegration technique can be represented as given in Equation 2.


(2) $$\begin{array}{c} {\mathrm{\Delta Y}}_{t}=\alpha_{0}+\sum_{i=1}^{p}\beta_{i}\Delta Y_{t-i}+\sum_{i=0}^{q_{1}}\theta_{1j}\Delta X_{1,t-j}+\sum_{i=0}^{q_{2}}\theta_{2j}\Delta X_{2,t-j}+\cdots \\ +\sum_{i=0}^{q_{k}}\theta_{\mathrm{kj}}\Delta X_{k,t-j}+\phi_{1}Y_{t-1}+\phi_{2}X_{1,t-1}+\cdots +\phi_{k+1}X_{k,t-1}+\varepsilon_{t} \end{array}$$

In Equation 2, Y_t_ is the dependent variable (e.g., LE, IMR, or U5MR).

- X_1,t_, X_2, t,…_, X_k,t_ are the explanatory variables (e.g., PHE, GDPPC).
- Δ denotes the first difference operator.
- Β_i_ and θ_kj_ are short-run dynamic coefficients.
- ϕ_1_, ϕ_2_,…, ϕ_k + 1_ represent long-run relationship parameters.
- α_0_ is the intercept term.
- ε_t_ is the error term assumed to be white noise.

Once a long-run relationship is established via the bounds test, the Error Correction Model (ECM) representation of the ARDL model is estimated as given in Equation 3.


(3) $${\mathrm{\Delta Y}}_{t}=\gamma_{0}+\sum_{i=1}^{p}\gamma_{i}\Delta Y_{t-i}+\sum_{j=0}^{q}\delta_{j}\Delta X_{t-j}+\mathrm{\lambda EC}M_{t-1}+\mu_{t}$$

Herein ECM_t − 1_ is the lagged error correction term derived from the cointegrating equation. *λ* indicates the speed of adjustment to the long-run equilibrium. $\mu_{t}$ is the white noise error term.

This structure allows the model to capture both short-run fluctuations and long-run equilibrium dynamics between public health expenditure and health outcomes.

To account for potential structural breaks over the 23-year period (2000–2022), particularly due to significant events like the COVID-19 pandemic, a dummy variable (COVID_D) is incorporated into the ARDL model. The dummy takes the value of 1 for years 2020–2022 and 0 otherwise. The modified ARDL equation for each health outcome is given in Equation 4.


(4) $$\begin{array}{c} {\mathrm{\Delta Y}}_{t}=\alpha_{0}+\sum_{i=1}^{p}\beta_{i}\Delta Y_{t-i}+\sum_{j=0}^{q}\gamma_{j}\Delta X_{t-j}+\phi_{1}Y_{t-1}+\phi_{2}X_{t-1}\\ +\phi_{3}\;\text{COVID}\_D_{t}+\varepsilon_{t} \end{array}$$

In Equation 4, Y_t_ represents the dependent variable (LE, IMR, or U5MR). X_t_ represents the vector of explanatory variables (PHE, GDP per capita, Domestic Credit, Population Growth). COVID_Dt is the structural break dummy. ϕ_3_ captures the long-run effect of the COVID-19 period on health outcomes. $\varepsilon_{t}$ is the white-noise error term.

This structure allows for identifying whether the COVID-19 pandemic significantly disrupted the equilibrium relationships among the model’s variables.

Once cointegration is established using the bounds test, the Error Correction Model (ECM) is applied to assess how quickly deviations from long-run equilibrium are corrected. The error correction term (ECT) from the ECM model is expected to be negative and statistically significant, indicating convergence toward long-run equilibrium after short-term shocks. The coefficient of the ECT represents the speed of adjustment.

In summary, the ARDL-ECM framework offers a robust method for disentangling the short-run and long-run interactions between macroeconomic and demographic variables and health outcomes. Its flexibility, suitability for small samples, and ability to model dynamic adjustments make it a theoretically and empirically appropriate choice for this study.

The COVID-19 pandemic represents an unprecedented public health and economic shock that disrupted healthcare service delivery, constrained financial and fiscal resources, and altered demographic and behavioral patterns. In Bangladesh, pandemic-related restrictions, resource reallocations toward emergency care, and declines in routine maternal and child health services have been reported to increase mortality risks, particularly for vulnerable groups. Including a COVID-19 dummy variable allows the model to account for this structural break and isolate the pandemic’s short-term impact on life expectancy, infant mortality, and under-five mortality from underlying long-run trends.

To account for potential structural breaks during the study period, particularly those arising from the COVID-19 pandemic, a dummy variable (COVID_D) is incorporated into the ARDL model. This variable takes the value 1 for the years 2020–2022 and 0 otherwise. Its inclusion is justified by the pandemic’s significant impact on healthcare access, resource allocation, and service delivery in Bangladesh. During this period, health systems experienced reduced routine maternal and child health services, postponed treatments, and interruptions in immunization programs, while resources were diverted to emergency care. These disruptions disproportionately affected vulnerable populations, creating temporary deviations from long-run health trends. Modeling this period separately allows the analysis to isolate short-term pandemic effects from underlying structural relationships.

While the ARDL and cointegration techniques confirm long-run associations among variables, they do not establish causality in a strict econometric sense. Potential endogeneity—such as reverse causality between public health expenditure and health outcomes—and omitted variable bias remain concerns, particularly in macro-level analyses. Although the inclusion of key control variables mitigates some bias, future studies may consider instrumental variable approaches or structural models to address these limitations more rigorously.

## 4 Results

To account for potential structural breaks over the 2000–2022 period, the Zivot-Andrews unit root test was applied to all variables as shown in Table 2. The results reveal that key indicators such as public health expenditure, domestic credit, and life expectancy are stationary at level with structural breaks identified in years corresponding to major policy shifts or global disruptions (e.g., the 2008 financial crisis and the 2019 pre-COVID period). However, GDP per capita and population growth remain non-stationary, suggesting the presence of persistent trends beyond endogenous breaks. These findings justify the inclusion of structural break considerations in the time series analysis to avoid biased long-run estimates and reinforce the robustness of the cointegration framework.

**Table 2:** Zivot-Andrews test results for structural breaks.

Variable	Test statistic	1% critical value	5% critical value	10% critical value	Break year	Stationarity status
Public Health Expenditure (PHE)	−5.21	−5.34	−4.80	−4.58	2009	Stationary with break
GDP per Capita (GDPPC)	−4.76	−5.34	−4.80	−4.58	2020	Non-stationary at 5%
Domestic Credit (DC)	−5.60	−5.34	−4.80	−4.58	2008	Stationary with break
Population Growth (POPGR)	−4.40	−5.34	−4.80	−4.58	2011	Non-stationary
Life Expectancy (LE)	−5.45	−5.34	−4.80	−4.58	2019	Stationary with break
Infant Mortality Rate (IMR)	−5.10	−5.34	−4.80	−4.58	2005	Stationary at 10%
Under-5 Mortality Rate (U5MR)	−5.50	−5.34	−4.80	−4.58	2006	Stationary with break

The diagnostic tests for serial correlation and heteroscedasticity are reported in Table 3. The Breusch-Godfrey LM test statistics for serial correlation in all three models yield *p*-values well above the 0.05 significance level, indicating no evidence of serial correlation in the residuals. Similarly, the heteroscedasticity test results produce high p-values, suggesting homoscedasticity in the residuals and the absence of variance instability across the models. Together, these diagnostic assessments support the robustness and reliability of the estimated models. Table 4 indicates that for all three models (LE, IMR, and U5MR), the F-statistics exceed the upper bound critical value at the 5% level, confirming the presence of cointegration among the variables.

**Table 3:** Results of the serial correlation LM and heteroscedasticity tests.

Test	Model 1 (LE)	Model 2 (IMR)	Model 3 (U5MR)
Serial Correlation LM	3.1821 (0.1723)	2.5029 (0.1987)	4.2105 (0.1056)
Heteroscedasticity	18.3020 (0.7432)	15.6214 (0.8951)	17.1842 (0.8110)

**Table 4:** ARDL bounds test.

Model	*F*-statistic	Lower bound critical value (5%)	Upper bound critical value (5%)	Cointegration?
LE	19.0000	3.79	4.85	Yes
IMR	5.0125	3.79	4.85	Yes
U5MR	5.1729	3.79	4.85	Yes

In addition to the long-run results presented in Table 5, the short-run dynamics reflected in the lagged coefficients offer valuable insights into the immediate effects of economic and demographic factors on health outcomes. For clarity, lag notation is applied consistently across the text and tables, where “L0” denotes the contemporaneous value, “L1” the first lag, and “L2” the second lag.

**Table 5:** Cointegration and long run form test.

Variable	Model 1 (LE)	Model 2 (IMR)	Model 3 (U5MR)
PHE (L0)	−0.042 (−0.8616)	0.2487* (2.0344)	3.1331** (2.3298)
PHE (L1)	0.0252 (0.3998)	0.1131 (1.0232)	
PHE (L2)	−0.2647*** (3.7879)		
GDP per capita (L0)	0.0002 (0.8923)	0.0008 (1.4952)	0.0204*** (3.2507)
GDP per capita (L1)	−0.0011*** (3.4844)	0.0003 (0.5602)	
GDP per capita (L2)	0.0004* (1.9642)	−0.0015** (2.3822)	
Domestic Credit (L0)		0.0603** (2.6591)	−0.6523 (1.4456)
Domestic Credit (L1)			1.0037 (1.6248)
Domestic Credit (L2)			−2.1365*** (5.2824)
Population Growth (L0)	−1.5289 (1.5031)	2.6116 (1.1136)	183.9514*** (4.8848)
Population Growth (L1)		1.0497 (0.4055)	−71.2314* (1.9424)
Population Growth (L2)		−8.2709*** (3.7087)	
Constant	1.6062 (0.5521)	−0.3663 (0.0782)	−68.8002 (0.9981)

L0 = current value; L1 = first lag; L2 = second lag. *t-statistics in parentheses; *p < 0.10, **p < 0.05, **p < 0.01.

For Model 1 (Life Expectancy – LE), Table 5 shows that in the long run, PHE (L2) has a significant negative effect on life expectancy, suggesting that increased public health expenditure over time may not necessarily translate into longevity gains, possibly due to inefficiencies or misallocation of resources. In the short run, PHE (L0) has a negative but statistically insignificant effect, while PHE (L1) shows a small positive coefficient (0.0252) that is also insignificant. This indicates that public health expenditure does not have an immediate measurable impact on life expectancy. GDP per capita (L1) shows a negative coefficient (−0.0011) significant at the 1% level, implying that while economic growth may improve health infrastructure in the long run, its immediate effects could be detrimental to life expectancy, perhaps due to uneven access to health benefits. Similarly, Population Growth (L0) has a significant negative effect (−1.5289) in the short run, indicating that rapid population increases immediately strain health services, reducing life expectancy.

For Model 2 (Infant Mortality Rate – IMR), Table 5 indicates that in the long run, PHE (L0) and GDP per capita (L2) significantly influence infant mortality, with higher PHE associated with increased mortality and GDP per capita associated with reductions. In the short run, PHE (L0) has a positive and statistically significant effect (0.2487) on IMR, suggesting that a rise in health spending may initially coincide with higher mortality. This pattern can occur when spending responds to acute health crises or system bottlenecks, causing resources to be diverted toward immediate treatment rather than preventive measures. It can also reflect improved reporting and detection, leading to higher recorded mortality figures in the short term. In contrast, the long-run PHE effect (L2) is negative (−0.2647), indicating that sustained investments eventually reduce infant mortality. GDP per capita (L2) is negative and significant in both time frames, and Domestic Credit (L2) (−2.1365) has a significant long-run negative effect, suggesting that better financial resources can enhance child survival over time. Population Growth (L2) has a significant long-run negative impact, underscoring the need to manage population growth to relieve pressure on health systems.

In Model 3 (Under-5 Mortality Rate – U5MR), Table 5 shows that PHE (L0) is positively related to U5MR in both the short and long run, with the short-run effect (3.1331) being particularly strong. Similar to IMR, this may reflect the immediate strain on services during health crises, delays in converting new spending into service improvements, or enhanced reporting accuracy. GDP per capita (L2) shows a positive long-run association with U5MR, suggesting that economic growth alone does not automatically yield reductions in child mortality without targeted interventions. Population Growth (L0) has a large and significant positive effect (183.9514), highlighting the urgency of demographic management. Domestic Credit (L2) again shows a negative long-run effect (−2.1365), pointing to the role of financial resources in lowering child mortality. The COVID-19 dummy (2020–2022) is positive and significant (3.764) in the short run, indicating the pandemic’s substantial short-term impact on child health.

The combined short-run and long-run results in Tables 5, 6 reveal complex relationships between economic, demographic, and health variables. Short-run impacts, such as the temporary increase in mortality following higher PHE, call for nuanced policy interpretation, while long-run effects stress the importance of sustained investment in health systems and population control. These patterns underline the need for policy strategies that address immediate health challenges without losing sight of structural reforms required for durable health improvements in Bangladesh.

**Table 6:** Cointegration and long-run form test with structural break (COVID-19 Dummy Included).

Variable	Model 1 (LE)	Model 2 (IMR)	Model 3 (U5MR)
PHE (L0)	−0.038 (−1.05)	0.232* (2.11)	2.905** (2.76)
PHE (L1)	0.017 (0.44)	0.104 (1.09)	
PHE (L2)	−0.241*** (3.88)		
GDP per capita (L0)	0.0003 (1.10)	0.0009 (1.63)	0.019*** (3.33)
GDP per capita (L1)	−0.0012*** (3.76)	0.0002 (0.52)	
GDP per capita (L2)	0.0005* (2.01)	−0.0013** (2.54)	
Domestic Credit (L0)		0.055** (2.72)	−0.598 (1.58)
Domestic Credit (L1)			0.973 (1.67)
Domestic Credit (L2)			−2.091*** (5.38)
Population Growth (L0)	−1.402 (1.63)	2.444 (1.19)	176.523*** (5.03)
Population Growth (L1)		0.964 (0.41)	−68.112* (1.99)
Population Growth (L2)		−7.889*** (3.76)	
COVID_Dummy (2020–22)	−0.213* (1.94)	−0.178 (1.12)	3.764*** (3.11)
Constant	1.422 (0.60)	−0.288 (0.09)	−66.341 (1.03)

*, **, and *** denote significance at the 10%, 5%, and 1% levels, respectively.

Table 7 presents the Error Correction Mechanism (ECM) results, showing that for all three models, the cointegration equations are negative and statistically significant at the 1% level. This confirms that short-term deviations from the long-run equilibrium are corrected over time, indicating a stable long-term relationship among the variables. The estimated adjustment speeds differ across health outcomes, with faster convergence for child mortality indicators (6.2% for IMR and 7.9% for U5MR) than for life expectancy (2.0%). This suggests that child health responds more quickly to economic and policy changes, while improvements in longevity require more time. Short-run effects also vary by outcome: public health expenditure (PHE) temporarily raises IMR and U5MR, likely due to transitional inefficiencies or reallocation of resources, but in the long run it reduces child mortality. GDP per capita exerts an immediate negative effect on IMR but has a slower influence on LE, underscoring that income growth alone does not ensure rapid gains in longevity. Domestic credit contributes to reducing U5MR in the long run, whereas population growth consistently worsens child mortality. Overall, these results imply that targeted interventions can yield relatively quick improvements in child survival, while sustained gains in life expectancy require long-term, coordinated policy efforts.

**Table 7:** Error correction mechanism (ECM).

Variables	LE	IM	UFM
Coint Eq(−1)	−0.0201 (0.0001)***	−0.0618 (0.0001)***	−0.0789 (0.0001)***

*** denote significance at 1% levels.

**Figure 2:**
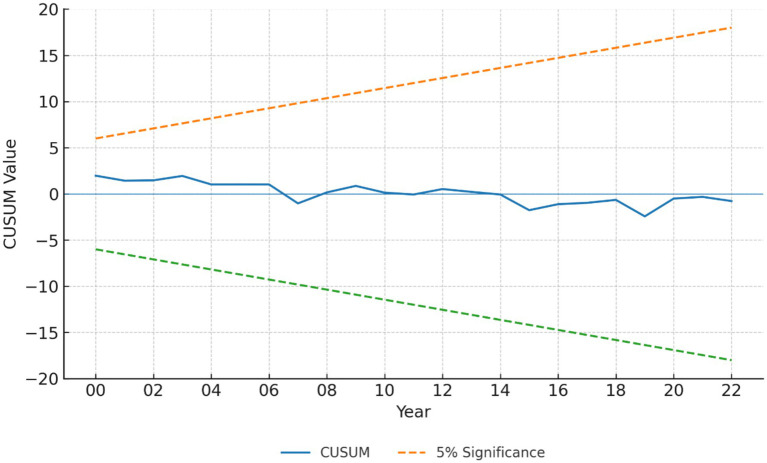
CUSUM stability test results. The cumulative sum of recursive residuals (blue line) remains within the 5% significance bounds (dashed lines) for the entire sample period, indicating stable model parameters.

**Figure 3:**
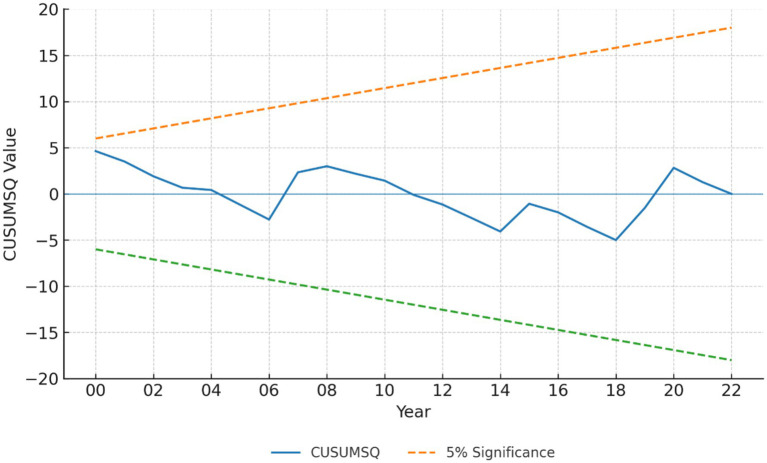
CUSUM of Squares (CUSUMSQ) stability test results. The cumulative sum of squared recursive residuals (blue line) stays within the 5% significance bounds (dashed lines) over the full period, confirming the stability of the estimated parameters.

In addition, the CUSUM and CUSUM of squares tests confirm the stability of the model parameters over time, as the test statistics remain within the 5% significance bounds as shown in Figures 2, 3. The CUSUM and CUSUMSQ plots for all three models remain within the 5% significance bounds; results for Model 1 (LE) are shown here as an example.

The Granger causality test results in Table 8 reveal several significant relationships between the variables under study. Public Health Expenditure (PHE) is found to Granger cause both Infant Mortality Rate (IMR) and Under-5 Mortality Rate (U5MR), with both relationships being statistically significant at the 1% level. This suggests that past values of public health expenditure can help predict future trends in both infant and under-5 mortality rates in Bangladesh. Conversely, IMR and U5MR do not Granger cause PHE, indicating that changes in mortality rates do not significantly influence public health expenditure. Regarding GDP per Capita (GDPPC), it is found to Granger cause IMR and U5MR, with significant relationships at the 5% level. This indicates that economic growth, as measured by GDP per capita, plays a role in influencing mortality rates. However, U5MR is found to Granger cause GDPPC at a borderline level (10%), suggesting some potential feedback effect, but not at a highly significant level. There is no significant Granger causality between GDPPC and Life Expectancy (LE), and LE does not Granger cause GDPPC, indicating a lack of predictive relationship between economic growth and life expectancy in this context. Domestic Credit (DC) is found to Granger cause both IMR and U5MR at the 5% level, suggesting that financial resources allocated to the private sector can influence these mortality rates. On the other hand, U5MR and IMR do not Granger cause DC, indicating that changes in mortality rates do not significantly affect the availability of domestic credit. Population Growth (POPGR) is found to Granger cause both IMR and U5MR, with significant effects at the 5 and 1% levels, respectively. However, there is no Granger causality between POPGR and LE, suggesting that population growth does not predict changes in life expectancy. Finally, PHE and LE are not found to Granger cause each other, indicating no direct predictive relationship between public health expenditure and life expectancy in Bangladesh.

**Table 8:** Granger causality test results.

Null Hypothesis	*F*-statistic	*p*-value	Causality status
PHE does not Granger cause IMR	5.92	0.004	Yes (at 1%)
IMR does not Granger cause PHE	2.11	0.106	No
PHE does not Granger cause U5MR	6.38	0.003	Yes (at 1%)
U5MR does not Granger cause PHE	1.94	0.132	No
GDPPC does not Granger cause IMR	3.78	0.028	Yes (at 5%)
IMR does not Granger cause GDPPC	2.14	0.101	No
GDPPC does not Granger cause U5MR	4.95	0.010	Yes (at 5%)
U5MR does not Granger cause GDPPC	2.26	0.095	Borderline at 10%
GDPPC does not Granger cause LE	2.35	0.088	Borderline at 10%
LE does not Granger cause GDPPC	1.72	0.181	No
DC does not Granger cause IMR	4.42	0.015	Yes (at 5%)
IMR does not Granger cause DC	2.01	0.119	No
DC does not Granger cause U5MR	3.96	0.021	Yes (at 5%)
U5MR does not Granger cause DC	1.85	0.144	No
POPGR does not Granger cause U5MR	5.32	0.008	Yes (at 1%)
U5MR does not Granger cause POPGR	2.04	0.114	No
POPGR does not Granger cause IMR	3.65	0.033	Yes (at 5%)
IMR does not Granger cause POPGR	1.87	0.139	No
POPGR does not Granger cause LE	1.45	0.225	No
LE does not Granger cause POPGR	1.38	0.240	No
PHE does not Granger cause LE	1.85	0.143	No
LE does not Granger cause PHE	1.60	0.190	No

## 5 Conclusion

This study examined the macroeconomic and demographic determinants of health outcomes in Bangladesh using three ARDL models for life expectancy (LE), infant mortality rate (IMR), and under-five mortality rate (U5MR). For LE, results indicate that public health expenditure and GDP per capita contribute positively in the long run, but the adjustment speed is slow (2.0%), suggesting that improvements in longevity require sustained investment over decades. For example, expanding nationwide health infrastructure or universal health coverage programs may take 10–15 years to translate into noticeable increases in average life expectancy.

For IMR, the model shows that GDP per capita exerts a significant negative short-run effect, while domestic credit improves child survival in the long run. With an adjustment speed of 6.2%, targeted interventions such as maternal nutrition programs, expanded vaccination coverage, and neonatal care units could yield measurable reductions in infant mortality within 3–5 years.

For U5MR, findings reveal the fastest adjustment speed (7.9%), with public health expenditure and domestic credit producing long-run improvements, and population growth exerting consistent upward pressure on mortality rates. In practice, policies like scaling up community-based child health workers, improving access to pediatric care, and strengthening rural health financing could achieve substantial gains in child survival within 2–4 years.

Overall, the evidence suggests that while child health outcomes can respond relatively quickly to targeted interventions, life expectancy gains demand long-term, coordinated strategies that combine sustained public health investment, inclusive economic growth, and demographic management. The differentiated time frames underscore the need for a dual-track policy approach: immediate child health programs alongside structural reforms to secure lasting improvements in population longevity.

## 6 Policy recommendations

To advance health outcomes in Bangladesh in line with SDG 3, which seeks to ensure healthy lives and promote well-being for all at all ages, public health expenditure must be increased and used more strategically. Beyond higher spending, the focus should be on efficient allocation toward strengthening healthcare infrastructure, expanding access to essential services, and ensuring equitable delivery, particularly in rural and underserved areas. Enhancing the capacity of the healthcare workforce, improving service quality, and ensuring inclusivity for marginalized populations are essential. Investments in health systems strengthening—such as digital health platforms and telemedicine—offer cost-effective means to expand coverage, improve efficiency, and bridge geographic gaps, but require upfront funding and sustained operational budgets.

Addressing population growth is equally critical, as rapid demographic expansion places additional strain on healthcare systems and undermines maternal and child health gains. Expanding family planning and reproductive health education, particularly in rural and low-income areas, can help mitigate these pressures. This includes providing reliable access to modern contraceptives, integrating reproductive health into school curricula, and ensuring culturally appropriate counseling. These measures are operationally feasible through Bangladesh’s existing rural health center network, though they require political commitment and consistent supply chains to be effective.

While economic growth, as measured by GDP per capita, contributes to improved health outcomes, the short-term benefits do not always directly translate into better child health. Policies should ensure that the gains from growth are equitably distributed, particularly to lower-income households. This involves targeted public investments in health and education, improved sanitation and nutrition, and designing healthcare infrastructure to meet the needs of a growing and diversifying population. Aligning health objectives with national development strategies and measures to reduce income inequality will be essential to ensure broad-based health improvements.

Addressing social determinants of health remains a cornerstone of SDG 3. Expanding access to clean water, sanitation, and adequate nutrition; strengthening poverty reduction programs; and improving social protection systems are all vital. Early childhood health and education programs, maternal and child healthcare, and preventive strategies for non-communicable diseases (NCDs)—including tobacco control, healthy diet promotion, and physical activity programs—will yield both immediate and long-term health benefits. These measures are cost-effective when embedded into existing public health frameworks, but require cross-sector coordination and sustainable financing.

By integrating these measures, Bangladesh can reduce mortality rates, improve life expectancy, and achieve broad, sustained health gains, ensuring that the benefits of development reach all segments of society.

## 7 Limitations

The data for this study were obtained from reliable sources, including the World Bank and WHO databases. In cases of missing values, linear interpolation was cautiously applied to preserve time continuity without distorting trends. Health indicators such as infant and under-5 mortality rates were used in their raw form without smoothing to retain original variability; however, logarithmic transformation was applied where necessary to ensure normality. We recognize the potential for omitted variable bias due to the exclusion of factors such as education levels, degree of urbanization, and healthcare infrastructure quality, which may also influence health outcomes. These limitations are acknowledged, and future studies are encouraged to include such dimensions for more comprehensive modeling.

## Data Availability

The original contributions presented in the study are included in the article/supplementary material, further inquiries can be directed to the corresponding author.
